# “In House” assays for the quantification of Annexin V and its autoantibodies in patients with recurrent pregnancy loss and in vitro fertilisation failures

**DOI:** 10.1038/s41598-023-49768-w

**Published:** 2023-12-15

**Authors:** Hossam Murad, Bouthina Ali, Aya Twair, Khaled Baghdadi, Marwan Alhalabi, Abdul Qader Abbady

**Affiliations:** 1 Division of Human Genetics, Department of Molecular Biology and Biotechnology, AECS, P. O. Box 6091, Damascus, Syria; 2 Division of Molecular Biomedicine, Department of Molecular Biology and Biotechnology, AECS, P. O. Box 6091, Damascus, Syria; 3 Division of Reproductive Medicine, Embryology and Genetics, Faculty of Medicine, Damascus University, Damascus, Syria

**Keywords:** Biological techniques, Biotechnology, Immunology, Molecular biology

## Abstract

Several studies have been shown that Annexin V (ANXV) autoantibodies concentrations are associated with both early recurrent pregnancy losses (RPLs) or in vitro fertilization failure (IVFf). We investigated the association between ANXV autoantibodies and ANVX levels in RPL, IVFf and normal group women. The study was conducted on 22 female patients with RPLs, 66 patients with IVFf, and 16 normal samples from women who had given birth. ANXV autoantibodies were measured using an ELISA test developed by fixing a homemade recombinant ANXV protein and examined with labeled human antibodies, while ANXV concentrations were measured by a competitive ELISA using a homemade anti ANXV polyclonal antibody. The results showed a clear relationship between the high levels of ANXV autoantibodies and the recurrent abortion. On the other hand, ANXV measurement in those patients showed decreased concentrations compared to normal samples. Negative correlation between ANXV and its autoantibodies levels was reported in almost all patients’ samples. Our data supports the possibility that ANXV autoantibodies are a risk factor for reproductive failures associated with both RPLs and/or IVFf and the significant role for ANXV in the maintenance of pregnancy.

## Introduction

Recurrent pregnancy losses (RPLs) happen to 1–5% of women around the world^1^. Parental genetic abnormalities, uterine anatomical defects, endocrine dysfunction, and hemostatic disorders are recognized as the known risk factors for RPL^2^. However, immunological risk factors are becoming more of a scientific focus. RPL patients are usually screened for antiphospholipid syndrome (APS), which is an autoimmune thromboinflammatory disorder, as recommended by most guidelines^3^. APS is classified according to the 2023 ACR/EULAR classification criteria by at least one positive antiphospholipid antibody (aPL), followed by additive weight criteria grouped into six clinical domains (macrovascular arterial thrombosis, macrovascular venous thromboembolism, microvascular, cardiac valve, obstetric and hematologic) and two laboratory domains (anticardiolipin [aCL] and/or anti–β2-glycoprotein I antibodies, and lupus anticoagulant functional coagulation assays)^4^. However, in daily practice APS may be much more complex and some patients have positive clinical signs associated with negative criteria aPL^5^. Non-criteria aPL such as (anti-vimentin/aCL and anti-phosphatidylserine/prothrombin) could cross the gap between APS and seronegative APS in diagnosis, prognosis, and treatment^6^.

Among aPL antibodies and antiphospholipid- binding proteins, autoantibodies directed toward Annexin V (ANXV) were considered as risk factors for recurrent miscarriage^7,8^.

ANXV, a Ca2^+^ and phospholipid binding glycoprotein, is present in various cells, tissues and in the bloodstream^9,10^. It was isolated and cloned from human umbilical cord arteries and placentae in the 1980s^11^. ANXV is famous as a placental anticoagulant protein I^12^, and has regulatory functions for inflammation and apoptosis^13^. ANXV is expressed by trophoblasts and endothelial cells and acts as an inhibitor of phospholipid-dependent blood coagulation reactions, its anticoagulant activity is associated with its high affinity for negatively charged phospholipids, especially, phosphatidylserine (PS) and its ability to act competitively with coagulation factors for the phospholipid-binding sites, preventing their activation^14^.

ANXV autoantibodies have been reported, but their role and their clinical correlates are controversial^15^. They were found in patients with arterial or venous thrombosis, especially in those with autoimmune rheumatic diseases such as systemic lupus erythematosus (SLE), primary APS or systemic sclerosis (SSc)^16^ and also found in patients with pregnancy loss with or without APS^15^. ANXV autoantibodies are suspected to exert a detrimental role and interfere with ANXV function. Thus, they have been associated with the occurrence of fetal loss and thrombosis during autoimmune^17^. Several studies suggest that displacement of ANXV shield from the syncytiotrophoblastic surface by ANXV autoantibodies is causative in the generation of thrombogenic environment and consequent fetal loss^18^. Monoclonal ANXV antibodies have the ability to remove ANXV from trophoblast cells' surface, resulting in a procoagulant effect^19^.

Previous work has demonstrated that ANXV and its autoantibodies can be measured in human plasma using enzyme linked immunosorbent assay (ELISA). The diagnostic significance of ANXV and its autoantibodies has been studied by many authors. However, the findings of these studies continue to be debatable^20^. No previously published reports have described plasma ANXV levels beside plasma ANXV autoantibodies levels in Syrian women with early RPLs or IVF failures. Therefore, these biological markers will be studied in Syrian patients to determine their relationship to miscarriage and their prognostic significance. We performed a study on healthy women without any history of thromboembolism, and RPL, IVFf patients. In addition, we evaluated the correlation between both the concentration of ANXV and its autoantibodies levels with miscarriage.

## Results

### Designing of ELISA detection tests

**Figure 1:**
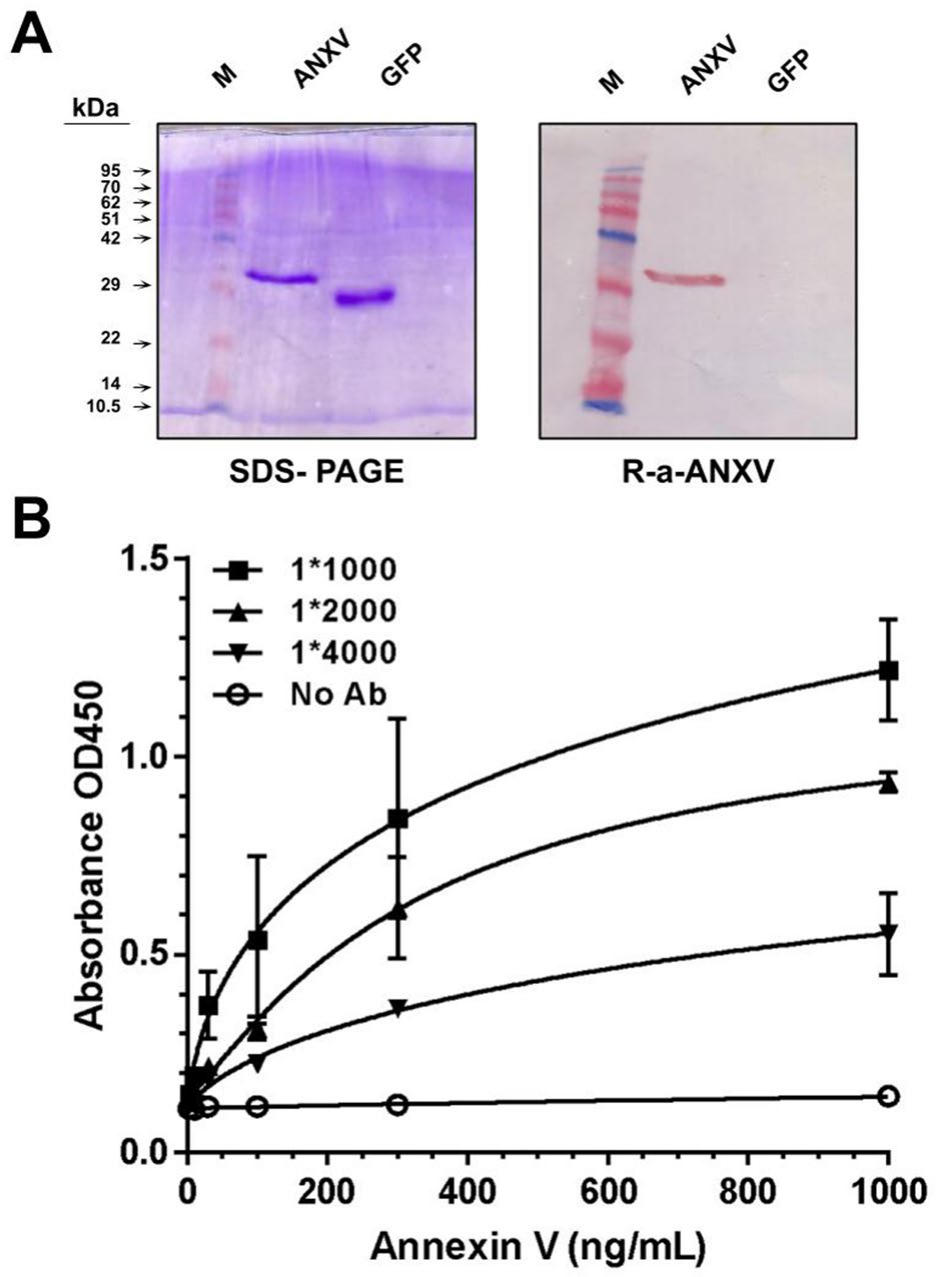
Preparation of rhANXV and its polyclonal antibody. (**A**) Purified proteins (0.5–2 µg/lane) were separated by SDS-PAGE then detected either by blue staining or by immunoblotting using rabbit anti-ANXV polyclonal antibody (R-a-ANXV/1:2000). Protein molecular weight ladder (M) (lane 1), rhANXV (lane 3), green fluorescent protein (GFP) was used as the negative control (lane 5), (line 2, 4 empty). (**B**) ELISA testing the reactivity of several dilutions (v:v) of R-a-ANXV antibody against serial concentrations (ng/mL) of immobilised rhANXV. Ab/Ag complexes were detected using goat anti-rabbit HRP-conjugated antibody (1:3000).

**Figure 2:**
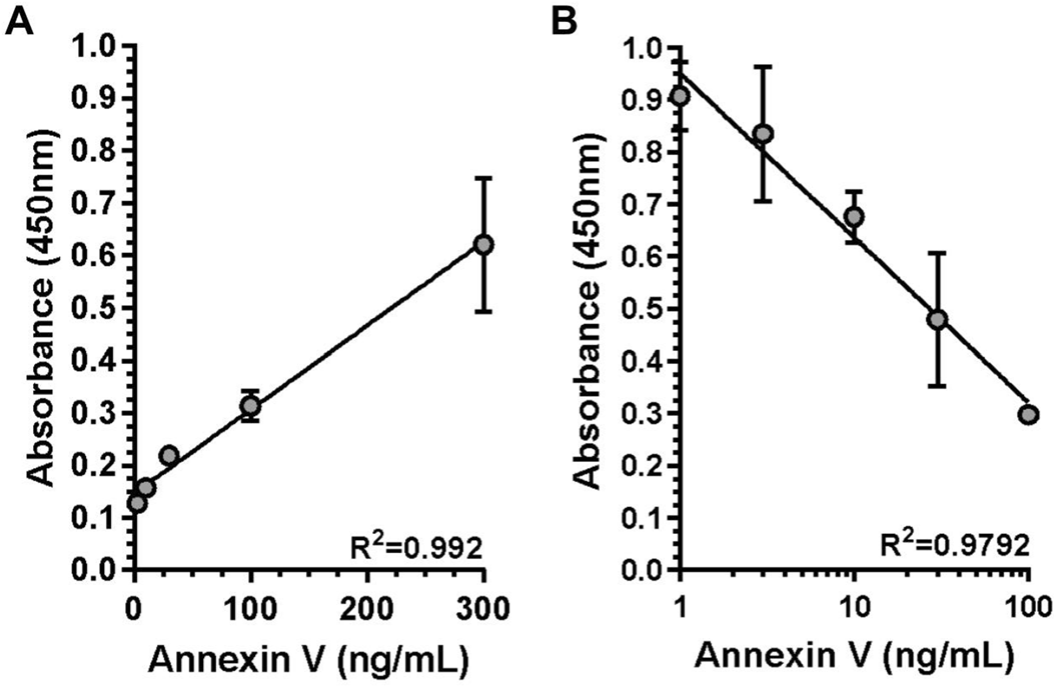
Testing the sensitivity of ANXV detection systems. Two ELISA systems were developed for quantitating ANXV and its autoantibodies levels. (**A**) Standard ELISA in pre-coated wells with serial concentrations (ng/mL) of immobilised rhANXV. (**B**) Competitive ELISA were performed after incubating R-a-ANXV (1:2000) with serial concentrations (ng/mL) of free ANXV then the mixture was added to the immobilised rhANXV (300 ng/mL). Ab/Ag complexes were detected using goat anti-rabbit HRP-conjugated antibodies (1:3000). Goodness of fit (R^2^) was shown for each curve.

Integrity of used rhANXV (36 kDa) was confirmed by SDS-PAGE separation followed either by blue staining or western blot using anti ANXV polyclonal antibody, and green fluorescent protein (GFP) was used as a negative control (Fig. 1A). The reactivity of several dilution of ANXV polyclonal antibody against serial concentration of immobilized rhANXV showed that 1/2000 was the optimal dilution of ANXV polyclonal antibody and (300 ng/ml) of immobilized rhANXV was suitable to be used in further experiments (Fig. 1B). For quantifying ANXV and its autoantibodies, we used two different ELISA systems. Standard ELISA was applied for the detection of ANXV autoantibodies in patients' plasma (Fig. 2A), while competitive ELISA was used to detect plasma ANXV levels (Fig. 2B). The two systems showed linear regression with high sensitivity and accuracy (R^2^ = 0.992, 0.9792) respectively.

### Detection of ANXV autoantibodies in RPL, IVFf patients

**Figure 3:**
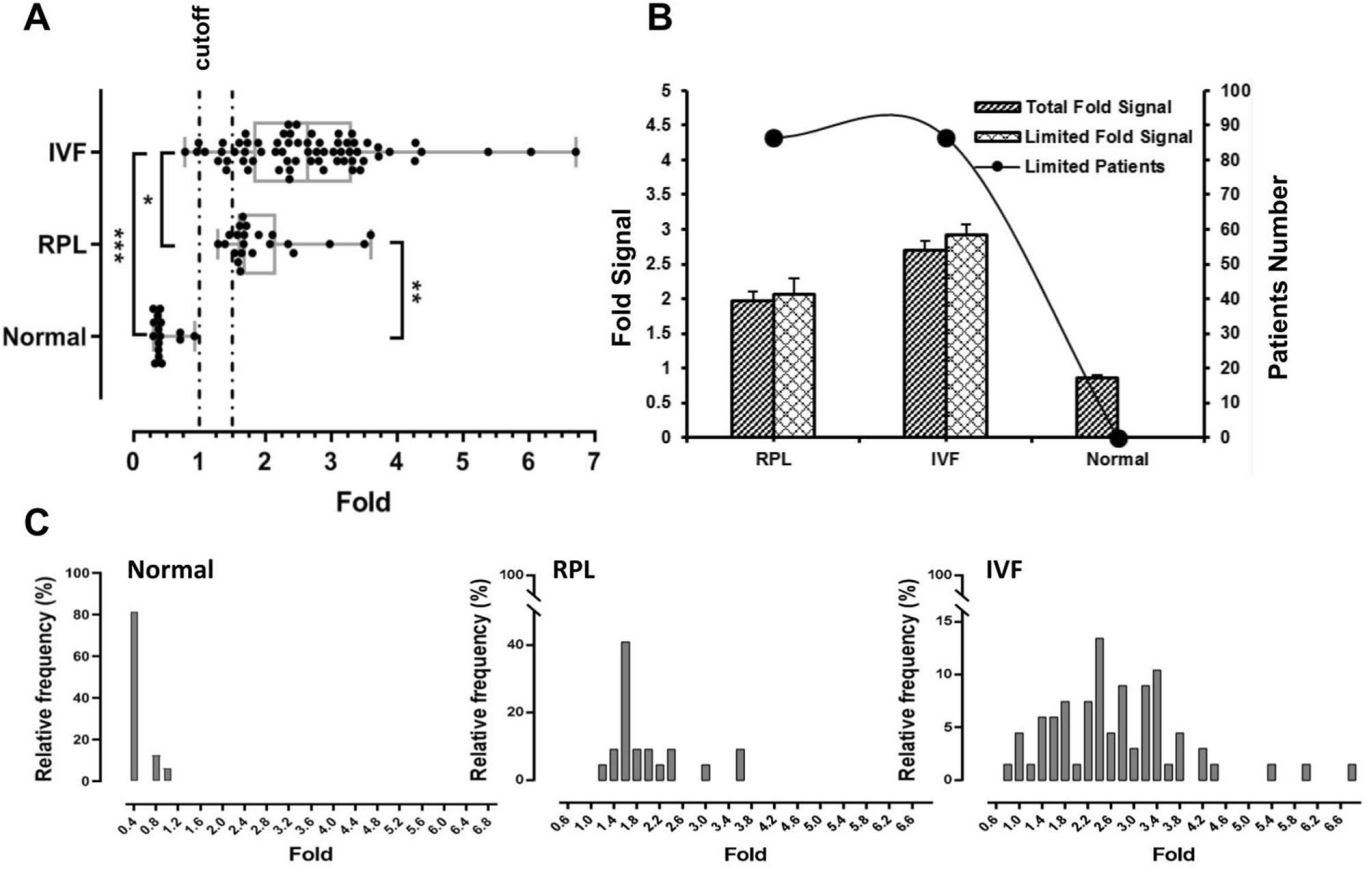
Detection of ANXV autoantibodies in RPL, IVFf patients. ANXV autoantibodies in the plasma of RPL, IVFf and normal samples were detected using standard ELISA. (**A**) Detection values were expressed as a fold increase in ELISA signal between rhANXV-coated wells (300 ng/mL) and uncoated blank wells. Dotted line defines the cutoff of positive samples (> 1.5 folds). (**B**) The means of fold signals for all samples (Total Fold Signal) in each group or for those in the group having detection value above the cutoff (Limited Fold Signal) are shown (Bars, left Y-axes). Percentage of samples having detection value above the cutoff for each group is shown (•, right Y- axes). (**C**) Relative frequencies (%) of patients according to their fold detection values in the different groups.

After applying plasma samples of RPL, IVFf and normal groups to standard ELISA. Normal controls were tested to establish a positive/negative cutoff value (1.5 fold). The net results of the ELISA tests expressed as OD values of uncoated control wells subtracted from OD values of rhANXV-coated wells. Results showed statistical significance increase in ANXV autoantibodies levels in RPL and IVFf patients compared with normal women (Fig. 3A). 90, 89% of IVFf and RPL patients respectively had high concentrations (above the cutoff value) of ANXV autoantibodies in comparison with normal samples (Fig. 3B). Representation of the relative frequency of patients by the ELISA fold signal values showed that more than 40% of RPL patients had high levels of ANXV autoantibodies compared to 75% of normal samples that had detection value below the cutoff. On the other hand, most of IVFf patients had detection values above the cutoff (between 1.4 and 4.2) (Fig. 3C).

### Detection of ANXV in RPL, IVFf patients

**Figure 4:**
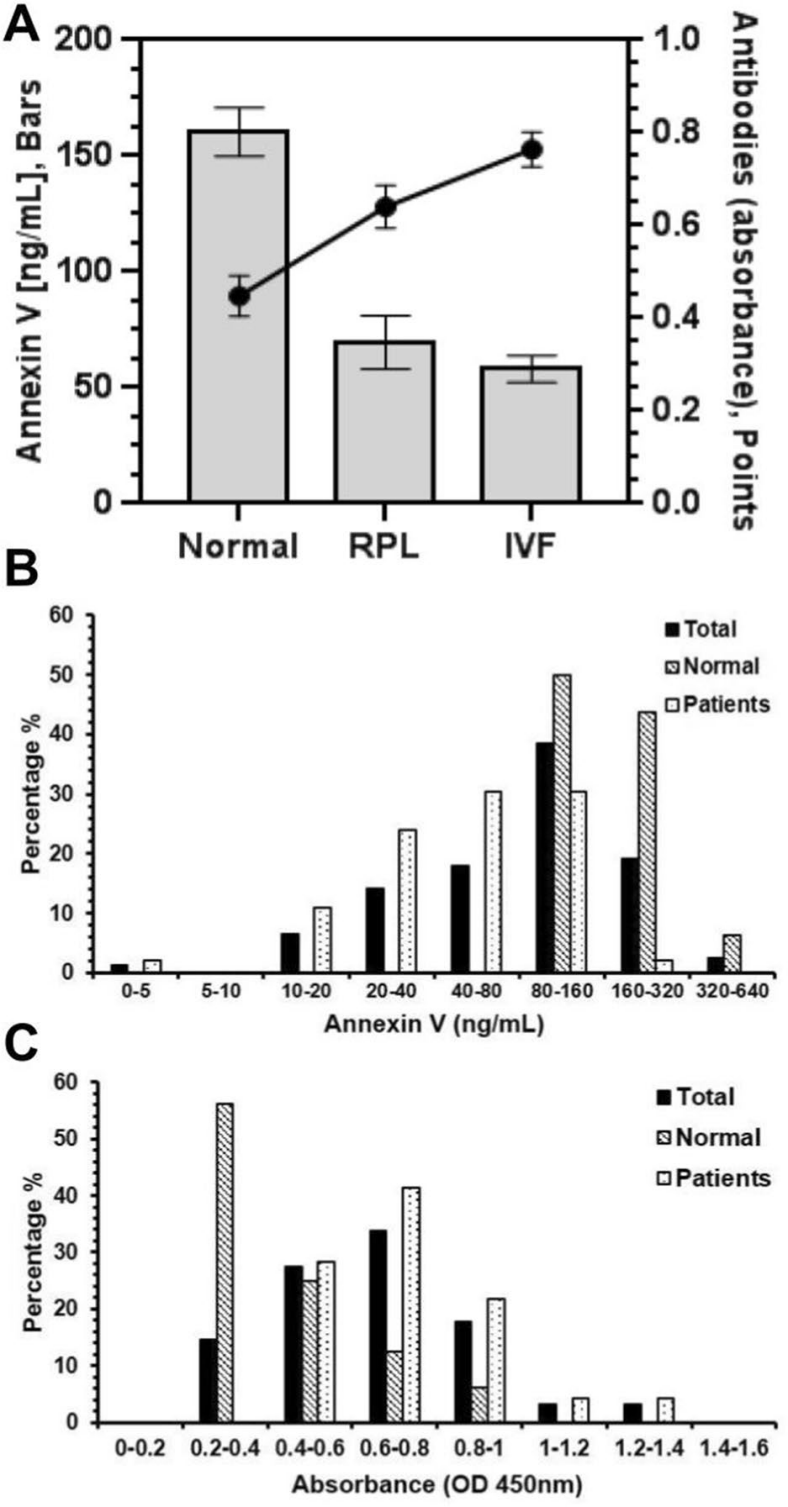
Detection of ANXV in RPL, IVFf patients. (**A**) The graph shows the average concentrations (ng/mL) of ANXV (Bars, left Y-axes), determined by competitive ELISA and ANXV autoantibodies absorbance detection signals (Points, right Y-axes), obtained by standard ELISA, for RPL, IVFf and normal samples. Percentage of total, normal and patient samples categorized in several ranges according to their ANXV concentration (ng/mL) (**B**) or ANXV autoantibodies absorbance (**C)**.

Concerning ANXV average concentrations, results by competitive ELISA showed decline in ANXV levels among RPL and IVFf women, 69, 57 ng/ml respectively in comparison to 160 ng/ml of ANXV in normal samples (Fig. 4A Bars). Conversely, ANXV autoantibodies absorbance detection signals obtained by standard ELISA, showed an elevation in ANXV autoantibodies in RPL and IVFf patients versus normal ones (Fig. 4A Curve). We also categorized samples in several ranges according to their ANXV concentration (ng/mL) or ANXV autoantibodies absorbance and results showed that most patients of RPL and IVFf had between 40 and 160 ng/ml of ANXV (Fig. 4B), and autoantibodies in range of 0.6–0.8 OD (Fig. 4C).

### Relationship between ANXV and its autoantibodies in patient samples

**Figure 5:**
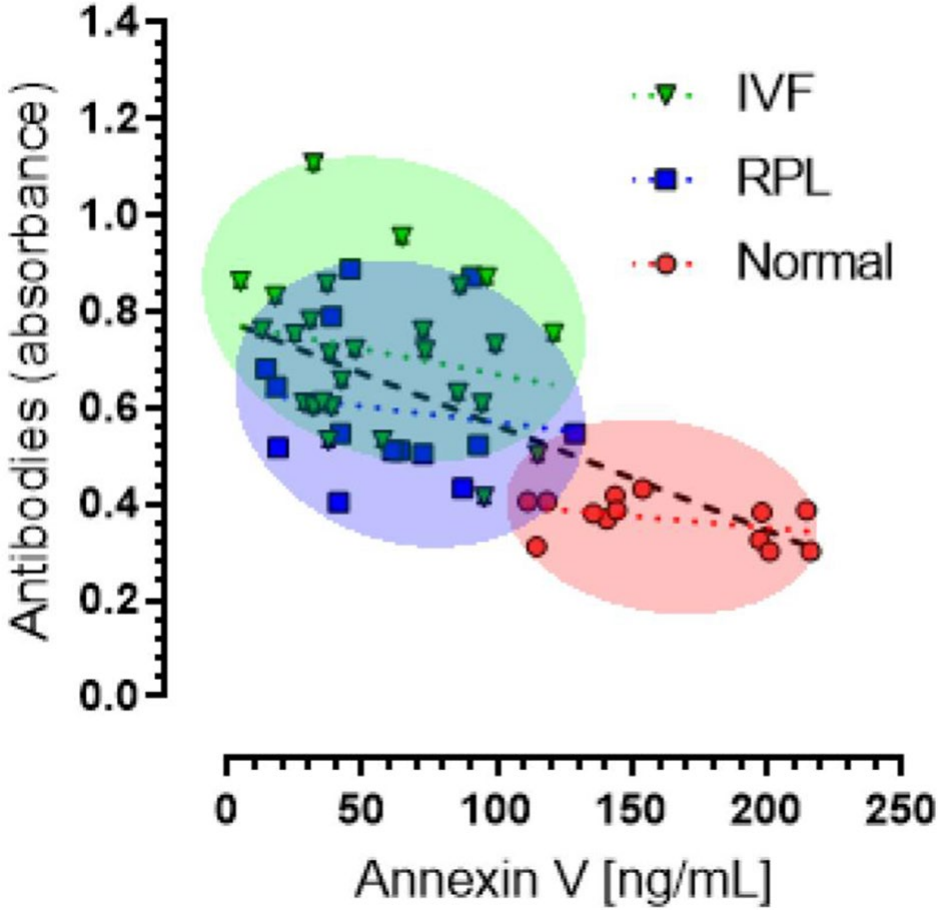
Relationship between ANXV and its autoantibodies in same patients. A scatter plot of ANXV concentration (ng/ml) and its autoantibodies levels (absorbance) of Normal (Red), RPL (Blue) and IVF (Green) samples. Analyses were performed using GraphPad Prism 9.

Within our cohort we observed a significant negative weak correlation between ANXV and its autoantibodies in the same patients (*P* < 0.0001, r = − 0.616). Pearson correlation coefficient was used to determine the r-value of the correlation between the two groups (Fig. 5).

## Discussions

In this work, an ELISA system was developed to study ANXV autoantibodies levels in RPLs and IVFf Syrian patients. Both RPLs and IVFf cases had a significantly increased incidence of anti ANXV IgG compared with normal nonpregnant women. Locally produced rhANXV was used in this work to develop the ELISA^21^. Assuming that ANXV is an APS cofactor, ANXV autoantibodies have been investigated in a number of clinical diseases. However, ANXV autoantibodies are of great interest in the gynecological field. Compared to other autoantibodies like aPL and anti-cofactor antibodies, ANXV antibodies proved to be an important risk factor associated with RPL in women with^22^ or without autoimmune disease^7,23^. Previous work has reported that recombinant and purified placental human ANXV give a similar ELISA signal while measuring ANXV autoantibodies levels^7^. ANXV autoantibodies were assessed as an independent risk marker for recurrent spontaneous abortion (RSA)^8^, and associated with combined early + late RSA in Tunisian women^24^. Similar results were concluded by Matsubayashi and colleagues^7^. Our data showed that ANXV autoantibodies were present in almost (90%) of RPLs and IVFf patients compared with normal control. ANXV has been described as forming a fetal maternal interface necessary to ensure successful pregnancy. This is supported by our data, which reveal that the presence of ANXV autoantibodies is a potential risk factor for reproductive failure.

ANXV is abundant in umbilical vein endothelial cells (4000 ng/mg total protein)^25^ and in placenta (250 mg/10 kg placenta)^26^. It is also present in amniotic fluid, seminal plasma and blood^27^. However, ANXV levels in normal human plasma are in the range of 0.3–151.5 ng/mL^28,29^. Due to its physiological roles, especially its anticoagulant activity, ANXV has been extensively studied. Several studies have shown change in ANXV levels in patients with trauma, cardiac arrest^30^, stroke^31^, systemic lupus erythematosus^32^, and primary antiphospholipid antibody syndrome^33^. Our study, the first to evaluate plasma ANXV levels in RPLs and IVFf Syrian patients, indicates that levels of this protein decreased in women with RPL or IVFf. *P* values of ANXV were < 0.05 indicating that there was a statistically significant difference (comparing RPL with normal or IVFf with normal). In addition, plasma ANXV levels show low negative correlation with the levels of its autoantibodies.

In Rand study, RPLs cases had significantly lower plasma ANXV levels than control subjects and significantly reduced anticoagulant ratios and reduced binding of ANXV to phospholipid, while there were no significant differences in ANXV autoantibody levels^28^. In addition, ANXV parameters do not distinguish between cases and controls when tested during pregnancy^29^. In addition, administration of ANXV into an animal model, inhibited thrombus formation and fibrin accretion in a related French study^34^.

The interaction between ANXV and antiphospholipid antibodies to the negatively charged phospholipids, like PS, has been reviewed by Rand et al.^35^ and discussed by Rote et al.^36^. Trophoblast externalizes PS in relation to differentiation that is required for appropriate placental growth^37^. PS supports the prothrombinase complex’s assembly when it is on the outside leaflet of a cellular membrane^38^. Extracellular ANXV recognizes this signal, binds to PS components of the membrane, and self-assembles laterally into an organized array. ANXV array forms a shield that prevents an excessive phospholipid-dependent coagulation reaction^39^, protects the integrity of the placental structure^40^ and thus is necessary for maintaining pregnancy. According to many reports, anti-PS or anti ANXV may displace ANXV from the trophoblast surface, exposing procoagulant membrane sites and triggering placental thrombosis, necrosis, and fetal loss^41^. In addition to these functions, more has to be learned about the physiological roles of ANXV and its autoantibodies^42^. Recently, anti ANXV positive cases were detected in the late COVID19 infection patients, suggesting a possible role for these anti-phospholipid antibodies in disease course. Nevertheless, ANXV was investigated as a potential treatment for inflammatory and hypercoagulable disorders. However, because it is rapidly removed after being infused into the circulation (t1/2, 15 min), its application is constrained^43^.

## Limitation of study

This study lacks information on other patient characteristics that can affect levels of annexin V and anti-annexin V, e.g., antiphospholipid antibody levels, medications, and comorbidities like diabetes and cardiovascular diseases.

## Conclusion

Our results accumulate once again with already available data to provide new evidence through samples from women in the Syrian community to prove the importance of diagnosing annexin V and its autoantibodies as a biomarker of miscarriages. In this work, the two developed ELISA could allow us to monitor this biomarker in a rapid, cheap and simple way especially when scanning a large number of samples. Our study demonstrates that women with RPL and recurrent IVFf showed a greater incidence of ANXV autoantibodies than normal nonpregnant women and this was correlated with decreased concentrations of plasma ANXV. These findings suggest that ANXV autoantibodies have an important function in infertility. In conclusion, ANXV autoantibodies proved to be biomarkers that are required for increasing diagnostic and risk-prediction of RPL and IVFf.

## Methods

### Patients and controls

All experimental protocols are approved by the Institutional Review Board of Atomic Energy community of Syria (AECS). All methods are conducted in compliance with the Declaration of Helsinki and its subsequent amendments. All methods were performed in accordance with the relevant guidelines and regulations. All patients were informed about the project of the study and asked to write a consent for blood sampling.

Blood samples were obtained with informed consent from 104 Syrian women (age, 31 years; range, 16–45 years), at the start of pregnancy with a documented positive β-hCG test or the presence of a gestational sac on ultrasound were recruited consecutively from Orient hospital in Damascus, Syria in the period of May 2017 to June 2018. Women were divided into three main groups; I. 22 RPL patients, II. Included 66 patients with recurrent IVFf, and III. 16 healthy women as a normal control group. RPL patients had two or more pregnancy losses (mean, 2.8; range, 2–7) before 10 weeks. Patients with recurrent IVFf had two or more failures despite good visual quality embryos. All healthy women delivered normally and denied previous pregnancy losses. The mean age of each group was 31 years (range, 22–42 years). Plasma was separated by two rounds of centrifugation at 1200 × g for 10 min at 4 °C to remove cellular contamination and supernatant was re-centrifuged at 16,000 × g for 10 min at 4 °C, and the supernatant was stored at − 80 °C.

### Preparation and detection of recombinant human ANXV

Recombinant human ANXV (rhANXV), with an N-terminal 6 × His tag, was prepared using the *E. coli* protein expression system as previously described^21^. The purity and integrity of rhANXV was confirmed by SDS-PAGE gel electrophoresis separation of ~ 2 μg of purified proteins on a 15% acrylamide gel, followed by blue staining, or western blotting under reducing conditions using homemade polyclonal rabbit anti rhANXV antibody (1:2000) followed by goat anti-rabbit horseradish peroxidase (HRP) (Bethyl laboratories) conjugated antibody (1:3000). Bands were revealed using the chromogen substrate AEC (3-amino-9-ethylcarbazole) prepared in acetate buffer containing H2O2.

### Production of polyclonal antibody against the recombinant rhANXV protein

Rabbit anti rhANXV polyclonal antibody was locally produced. One white female rabbit was immunized with three injections of purified rhANXV protein (500 µg) using standard procedure. After 45 days the immune response was evaluated by ELISA and rhANXV polyclonal antibody (IgGs) was purified from rabbit serum following the same affinity chromatography protocol as described in our previous work for different polyclonal antibodies^44–46^.

### Detection of ANXV autoantibodies in patients

Maxisorp 96-well plates were coated overnight with 100 μl rhANXV (300 ng/well) in a carbonate-bicarbonate buffer. After coating, ELISA plates were washed 3 times using 200 μl of washing buffer (1 × PBS containing 0.05% Tween-20). Residual protein binding sites in the wells were blocked for one hour at room temperature (RT) with a blocking buffer (1% bovine serum albumin (BSA), 3% skimmed milk in 1 × PBS). After the removal of the blocking buffer, 100 μl of rabbit anti rhANXV (1:2000) or a (1:1) dilution of patient or normal plasma, diluted in 1% blocking buffer, were added per well (100 μl) for one hour at RT. After 3 washes, detection of ANXV antibodies was performed by goat anti-rabbit (Bethyl laboratories) or anti-human IgGs (Bethyl laboratories) conjugated to HRP diluted (1:3000) in 1% blocking buffer. After an additional 3 washes, bound conjugate was revealed with 3,3′,5,5′-Tetramethylbenzidine (TMB) substrate (Sigma), the reaction was stopped after eight minutes by adding 50 µl of 1 M H_2_SO_4_. The OD at 450 nm was measured by Multiskan™ FC Microplate reader (Thermo Scientific™).

### Detection of ANXV within patients

A competitive ELISA investigated ANXV concentrations in normal or patient plasma samples. For this purpose, 50 µl of the patient's plasma was incubated individually with rabbit anti rhANXV (1:3000) for 1 h before adding them into a microtiter plate precoated with rhANXV (300 ng/ml). The wells were washed and the detection was performed with a polyclonal goat anti rabbit IgG labelled with HRP (1:3000) in a similar way as the previous ELISA.

### Statistical analysis

Data obtained were computerised using GraphPad Prism version 9. One-way ANOVA test was used for evaluating statistical significance for results. P value of less than 0.05 was considered to indicate statistical significance. The correlation between ANXV and its autoantibodies levels in different groups were determined using Pearson’s correlation coefficient (“Supplementary information”).

### Supplementary Information

Supplementary Information 1.

Supplementary Information 2.

## Data Availability

All data generated or analyzed during this study are included in the manuscript.

## Abbreviations

ANXV: Annexin V
PS: Phosphatidylserine
ELISA: Enzyme–linked immunosorbent assay
RPL: Recurrent pregnancy loss
IVFf: In vitro fertilization failure
