# Supplementary figures and images for: “In House” assays for the quantification of Annexin V and its autoantibodies in patients with recurrent pregnancy loss and in vitro fertilisation failures
Source: Sci Rep. 2023 Dec 15;13:22322. doi: 10.1038/s41598-023-49768-w (PMC10724132; doi:10.1038/s41598-023-49768-w)

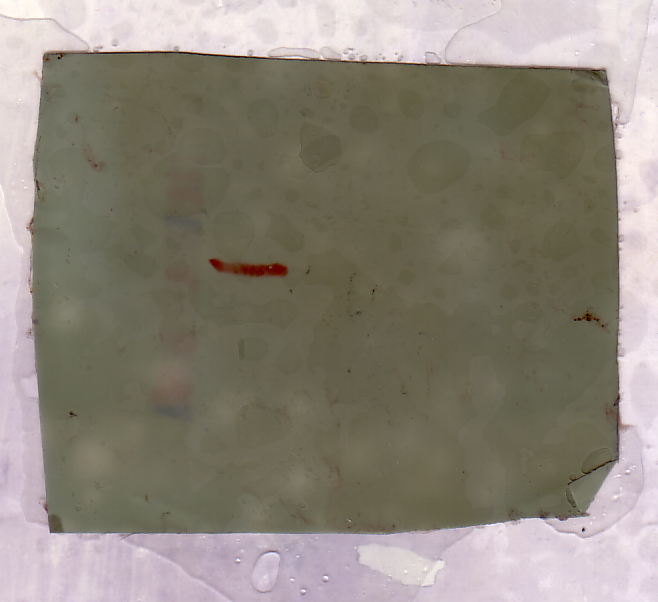

Supplement: Supplementary file 1 — Supplementary Information 1. [file 41598_2023_49768_MOESM1_ESM.jpg]

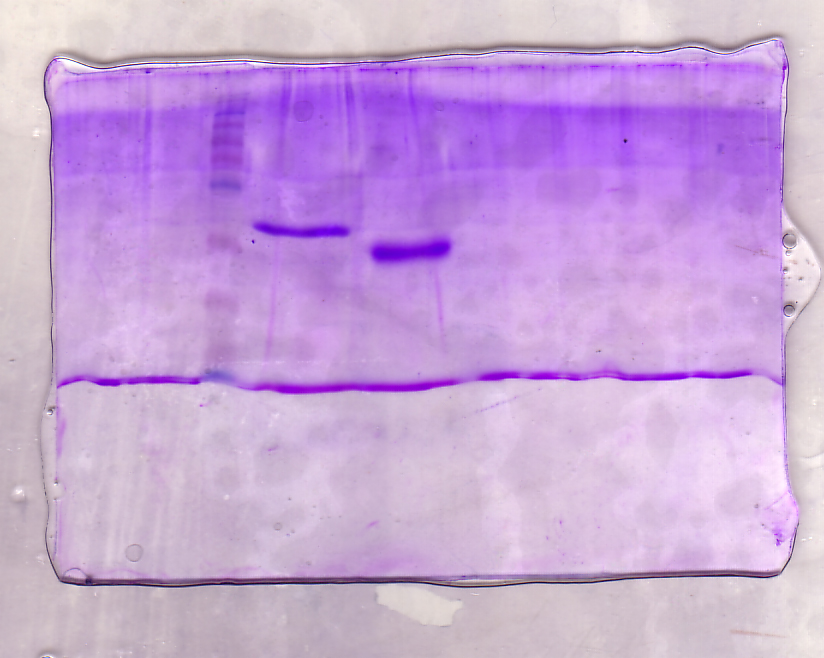

Supplement: Supplementary file 2 — Supplementary Information 2. [file 41598_2023_49768_MOESM2_ESM.jpg]
